# TMPYP4 exerted antitumor effects in human cervical cancer cells through activation of p38 mitogen-activated protein kinase

**DOI:** 10.1186/s40659-017-0129-4

**Published:** 2017-07-03

**Authors:** Ming-Jun Cheng, Yun-Gui Cao

**Affiliations:** Department of Gynaecology, Shanghai Jiading District Maternal and Child Care Hospital, No. 1216, Gaotai Road, Jiading District, Shanghai, 201821 China

**Keywords:** TMPyP4, p38 MAPK, Human cervical cancer cells, Proliferation, Apoptosis

## Abstract

**Background:**

The aim of the present study was to investigate the potential effects of the 5,10,15,20-tetrakis (1-methylpyridinium-4-yl) porphyrin (TMPyP4) on the proliferation and apoptosis of human cervical cancer cells and the underlying mechanisms by which TMPyP4 exerted its actions.

**Results:**

After human cervical cancer cells were treated with different doses of TMPyP4, cell viability was determined by 3-(4,5-dimethyl-2-thiazolyl)-2,5-diphenyl-2-H-tetrazolium bromide (MTT) method, the apoptosis was observed by flow cytometry (FCM), and the expression of p38 mitogen-activated protein kinase (MAPK), phosphated p38 MAPK (p-p38 MAPK), capase-3, MAPKAPK2 (MK-2) and poly ADP-ribose polymerase (PARP) was measured by Western blot analysis. The analysis revealed that TMPyP4 potently suppressed cell viability and induced the apoptosis of human cervical cancer cells in a dose-dependent manner. In addition, the up-regulation of p-p38 MAPK expression levels was detected in TMPyP4-treated human cervical cancer cells. However, followed by the block of p38 MAPK signaling pathway using the inhibitor SB203580, the effects of TMPyP4 on proliferation and apoptosis of human cervical cancer cells were significantly changed.

**Conclusions:**

It was indicated that TMPyP4-inhibited proliferation and -induced apoptosis in human cervical cancer cells was accompanied by activating the p38 MAPK signaling pathway. Taken together, our study demonstrates that TMPyP4 may represent a potential therapeutic method for the treatment of cervical carcinoma.

## Background

Cervical cancer is the fourth common malignant tumor in women which leads to approximately 274,000 mortalities every year worldwide according to the reports of the World Health Organization (WHO) [1]. Notably, 85% of cases and deaths occur in low- and middle-income countries [2]. Human papillomavirus (HPV) types is recognized as an essential precursor to the development of cervical cancer. The WHO has recommended the routine HPV vaccination in national immunization programmes worldwide. Early stage cervical cancer may be treated by triggering tumor cell apoptosis through the combined application of radiotherapy and chemotherapy [3]. However, patients with late-stage cervical cancer exhibit a poor physical condition, resulting in the limits of the application of radiotherapy, chemotherapy or the two therapies combined [4]. Currently, the pathogenesis of cervical cancer has not yet been completely understood, and there are no drugs available for effectively controlling the occurrence and development of this cancer [5]. So, it is urgent for us to seek new potential drugs and biomarkers for its diagnosis, prognosis, and therapy to improve clinical strategies of cervical cancer.

The cationic porphyrin, 5,10,15,20-tetra-(*N*-methyl-4-pyridyl) porphine (TMPyP4), a novel type of synthetic water-soluble photosensitizer, has been recently developed as a chemotherapeutics drug for treating cancers [6]. It has been reported that TMPyP4 leads to the arrest of tumor cell growth, and induces the apoptosis of tumor cells through reducing the telomerase activity [7–9], indicating that TMPyP4 presents a potential therapeutic target in tumor cells. Therefore, it is crucial to comprehensively understand biological effects of TMPyP4 in tumor cells before it can be used for anti-cancer therapeutics. In the present study, we evaluated the effects of TMPyP4 on the proliferation and apoptosis of human cervical cancer cells and further explored its underlying mechanisms.

## Methods

### Cell culture

Human cervical cancer cell line Hela and human normal cervical cells (Academia Sinica Cell Bank, Shanghai, China) were grown in low-glucose Dulbecco’s modified Eagle medium (GibcoBRL, Grand Island, NY, USA) supplemented with 10% (v/v) fetal bovine serum (Sigma-Aldrich Chemicals, USA), 100 IU/mL penicillin, and 10 mg/mL streptomycin. Cells were cultured in a incubator with 5% CO_2_ at 37 °C.

### Cell viability assay

Cell viability was assessed using MTT assay (Bestbio Biotechnology, Shanghai, China). Briefly, fresh human cervical cancer cells and human normal cervical cells at a concentration of 5 × 10^3^ cells/well were seeded in 96-well flat-bottomed tissue culture plates (Corning Inc., Corning, NY, USA) with complete culture medium and incubated for 24 h. Following two washes with phosphate-buffered saline (PBS), cells were incubated in 100 μL culture medium containing 1, 5, 10 or 20 μM TMPyP4 for 24 h at 37 °C prior to the MTT assay. Then, a total of 10 μL MTT and 100 μL culture medium was added to each well, and incubated for 1 h at 37 °C. The optical densities of the samples were measured directly using a spectrophotometric microplate reader (Beyotime Institute of Biotechnology, Haimen, China) at a wavelength of 490 nm. Each experiment was performed in triplicate and repeated six times.

### Cell apoptosis assay

The apoptotic cells were identified by FCM according to the published article [10]. Human cervical cancer cells and human normal cervical cells at a density of 2 × 10^4^/mL were cultured in 10% FBS-containing DMEM with 1, 5, 10 or 20 μM TMPyP4 for 24 h, respectively. Cells were harvested and washed twice with cold PBS by gentle shaking. Resuspended cells were added to 1× binding buffer and cell density was adjusted to 200,000–500,000/mL. In the dark, 5 μL of Annexin V-FITC (50 mM TRIS, 100 mM NaCl, 1% BSA, 0.02% sodium azide, pH 7.4) was added to the cell suspension in a mix of 195 μL and incubated for 10 min at room temperature before adding 190 μL 1× binding buffer and 10 μL propidium iodide (PI). Cell apoptosis was assayed using a FACScan flow cytometry apparatus (BD Biosciences, San Jose, CA, USA) and the percentage of apoptotic cells was analyzed using FlowJo 7.6.1 software (TreeStar, Inc., Ashland, OR, USA). Each experiment was performed in triplicate and repeated six times.

### Western blot analysis

Following the treatment with 20 μM TMPyP4 for 24 h, cells were collected for protein extraction. The examination of the expression levels of caspase-3, MK-2, PARP, p38 MAPK and p-p38 MAPK was then performed. Total protein was extracted and the bicinchoninic acid assay kit (Beyotime Institute of Biotechnology) was used to measure the protein concentration. A total of 20 μg protein was separated by SDS-PAGE and transferred onto a polyvinylidene fluoride membrane using wet transfer apparatus (Bio-Rad, Hercules, CA, USA). The membranes were then blocked with 5% skimmed milk and incubated overnight at 4 °C with the primary antibodies, followed by incubation with the secondary antibodies labeled with HRP. Next, the protein bands were visualized using an enhanced chemiluminescence kit (Millipore, Billerica, MA, USA) and the protein levels were detected using the chemiluminescence reader, ImageQuant™ LAS4000 (GE Healthcare, Pittsburgh, PA, USA). Antibodies were purchased from Santa Cruz Biotechnology, CA, USA. Band density was quantitated using Image J software (Image J 1.35, National Institute of Mental Health, USA).

### Caspase-3 activity assay

Caspase-3 activity was analyzed using caspase-3 activity assay kits according to the manufacturer’s instructions. Briefly, the reaction buffer and the specific enzyme DEVD-pNA were added to each cell plate and further cultured in an incubator for 1 h at 37 °C. The developed colorimetric reaction was measured at 405 nm in a 96-well Biorad 680 microplate reader (Bio-Rad, Hercules, CA, USA).

### Block of p38 MAPK signaling using inhibitor

Cells were treated with p38 MAPK inhibitor SB203580 (5 μM) for 24 h followed by addition of 20 μM TMPyP4, and further incubated for 24 h. Then, cell proliferation and apoptosis were evaluated.

### Statistical analysis

All results were analyzed using SPSS 17.0 statistical software (IBM, Armonk, NY, USA). Data are presented as the mean ± standard deviation (SD). Student’s two-tailed t test was used to determine the statistical differences between the treatment and control groups. P values were based on the two sided statistical analysis, and P < 0.05 was considered to indicate a statistically significant difference.

## Results

### TMPyP4 inhibited the proliferation rates of human cervical cancer cells

**Fig. 1:**
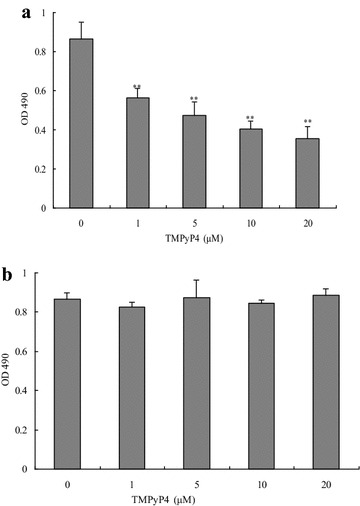
TMPyP4 inhibited cell proliferation in human cervical cancer cells. After cells were treated with 1, 5, 10 or 20 μM TMPyP4 for 24 h, cell proliferation in human cervical cancer cells (**a**) and human normal cervical cells (**b**) was measured by MTT assay. Data are expressed as mean ± SD of three independent experiments in six replicates. *P < 0.05 or **P < 0.01 indicates significance, *P* > 0.05 means no difference

To investigate the roles of TMPyP4 in human cervical cancer cells, we performed the MTT assay to evaluate the changes of cell viability. It was found that TMPyP4 (IC_50_ = 16.35 μM) significantly decreased OD_490_ values of human cervical cancer cells in a dose-dependent manner compared with the control group without TMPyP4 treatment (*P* < 0.05) (Fig. 1a). On the other hand, OD_490_ values of human normal cervical cells were minimally affected after exposed to TMPyP4 at different concentrations (*P* > 0.05) (Fig. 1b). These results indicated that TMPyP4 had low cytotoxic effects on human normal cells and inhibited cell proliferation in human cervical cancer cells.

### TMPyP4 induced the apoptosis of human cervical cancer cells

**Fig. 2:**
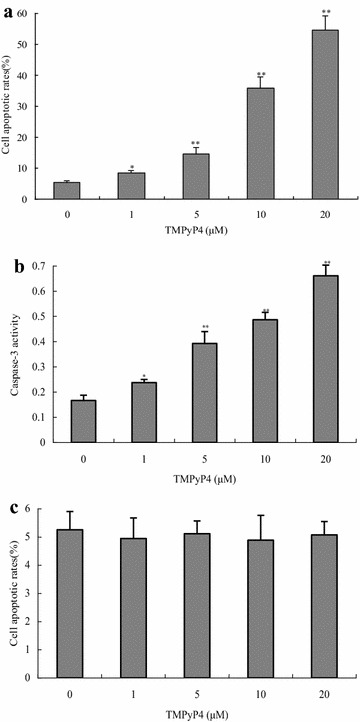
TMPyP4 induced cell apoptosis in human cervical cancer cells. Human cervical cancer cells were treated with 1, 5, 10 or 20 μM TMPyP4 for 24 h, the apoptotic rates (**a**), and caspase-3 activity (**b**) in cells was determined. The apoptotic rates of human normal cervical cells treated with 1, 5, 10 or 20 μM TMPyP4 for 24 h were tested by FCM (**c**). Data are expressed as mean ± SD of three independent experiments in six replicates. *P < 0.05 or **P < 0.01 indicates significance, *P* > 0.05 means no difference

To evaluate the apoptotic effects of TMPyP4 on human cervical cancer cells, cells were respectively exposed to different concentrations of TMPyP4 for 24 h. As shown in Fig. 2a, TMPyP4 remarkably induced the apoptosis of human cervical cancer cells in a dose-dependent manner. Approximately 9.0, 15.0, 36, 55.0% of cancer cells occurred apoptosis respectively after exposed to 1, 5, 10, 20 μM TMPyP4 for 24 h, but less than 6.0% of cancer cells without TMPyP4 stimulation showed apoptosis. Next, to further ascertain the apoptotic effects of TMPyP4 on human cervical cancer cells, typical apoptotic marker caspase-3 activity was measured by commercial kits. As shown in Fig. 2b, caspase-3 activity was promoted in TMPyP4-treated human cervical cancer cells (*P* < 0.05), which indicated that TMPyP4 indeed triggered apoptosis in human cervical cancer cells. Nevertheless, results in Fig. 2c provided the evidence that there was no significant apoptosis in human normal cervical cells after being exposed to the indicated concentration of TMPyP4 for 24 h (*P* > 0.05). These findings suggested that TMPyP4 obviously induced apoptosis in human cervical cancer cells, but not in human normal cervical cells.

### TMPyP4 activated p38 MAPK signaling in human cervical cancer cells

**Fig. 3:**
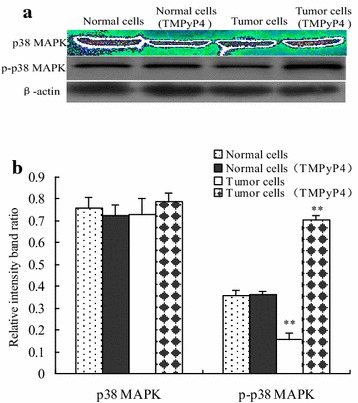
TMPyP4 activated the p38 MAPK signaling pathway in human cervical cancer cells. Human cervical cancer cells or human normal cervical cells were treated with 20 μM TMPyP4 for 24 h, the protein expression level of p38 MAPK and p-p38 MAPK was measured by Western blot (**a**). Band density was analyzed using Image J software (**b**). Data are expressed as mean ± SD of three independent experiments in triplicate. *P < 0.05 or **P < 0.01 indicates significance, *P* > 0.05 means no difference

To determine the mechanisms underlying the promotion of cell apoptosis, we assessed the expression pattern of p38 MAPK signaling components including p38 MAPK and p-p38 MAPK by Western blot assay in human normal cervical cells and human cervical cancer cells (Fig. 3a). Western blot analysis of p38 MAPK found that p38 MAPK protein was expressed in human cervical cancer cells and human cervical cancer cells, but it showed no difference between TMPyP4 (20 μM) treated cells and non-TMPyP4 treated cells (*P* > 0.05). Furthermore, there was no obvious changes in protein expression of p-p38 MAPK between TMPyP4-treated normal cells and the control normal cells (*P* > 0.05). In contrast, the p-p38 MAPK expression level was comparatively low in human cervical cancer cells compared with normal cells, and elevated protein level of p-p38 MAPK was demonstrated in TMPyP4-treated cells in comparison with cancer cells without TMPyP4 stimulation (*P* < 0.05) (Fig. 3b). It was suggested that TMPyP4 could activate the p38 MAPK signaling pathway in human cervical cancer cells.

### Activation of p38 MAPK signaling in human cervical cancer cells is required for TMPyP4-induced down-regulation of cell viability

**Fig. 4:**
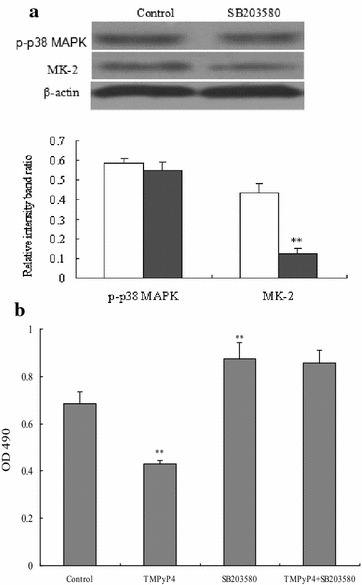
The p38 MAPK signaling pathway was involved in TMPyP4-inhibited cell proliferation in human cervical cancer cells. Cells were seeded in 6-well plates for 48 h and then treated with SB203580 in human cervical cancer cells for 24 h, Western blot was performed to determine protein expression of p-p38 MAPK and MK-2 for evaluating the blocking efficiency of SB203580 (**a**). Cells were treated with SB203580 for 24 h, and 20 μM TMPyP4 was added for 24 h, then cell viability was determined by MTT method (**b**). Data are expressed as mean ± SD of three independent experiments in six replicates. *P < 0.05 or **P < 0.01 indicates significance, *P* > 0.05 means no difference

Further, we examined the influence of p38 MAPK signaling on TMPyP4-induced down-regulation of cell viability in human cervical cancer cells. Cells treated with p38 MAPK signaling inhibitor SB203580 showed a decline in MK-2 protein expression, but had no effects on p-p38 MAPK expression compared to the control cells (Fig. 4a), suggesting better inhibitory efficiency of SB203580. Cell viability was promoted showing as high OD_490_ values in SB203580-treated cells while it was inhibited in TMPyP4-treated cells compared to the control (*P* < 0.05). However, there was no difference in cell OD_490_ values between SB203580-TMPyP4 co-treated cells and SB203580 treated cells (*P* > 0.05) (Fig. 4b).

### Activation of p38 MAPK signaling in human cervical cancer cells is required for TMPyP4-triggered apoptosis

**Fig. 5:**
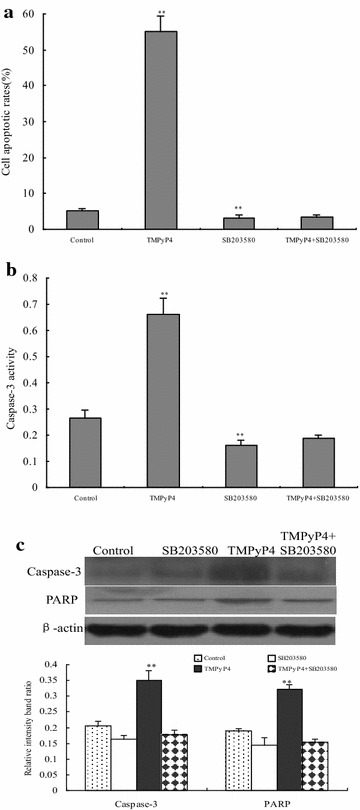
The p38 MAPK signaling pathway was involved in TMPyP4-triggered cell apoptosis in human cervical cancer cells. Cells were treated with SB203580 for 24 h, and 20 μM TMPyP4 was added for 24 h, then cell apoptotic rates (**a**), caspase-3 activity (**b**), PARP and caspase-3 protein expression (**c**) were measured. Data are expressed as mean ± SD of three independent experiments in six replicates. *P < 0.05 or **P < 0.01 indicates significance, *P* > 0.05 means no difference

To further confirm whether p38 MAPK activation contributed to TMPyP4-triggered apoptosis in human cervical cancer cells, a pharmacological inhibitor of p38 MAPK, SB203580 was used in this study. It was found that pretreatment of 5 μM SB203580 remarkably attenuated the apoptotic rate (Fig. 5a) and caspase-3 activity (Fig. 5b), while TMPyP4 significantly induced apoptosis by promoting the apoptotic rate, caspase-3 activity, and PARP and caspase-3 protein expression (Fig. 5c) in human cervical cancer cells (*P* < 0.05). Moreover, the apoptotic rate, caspase-3 activity, caspase-3 and PARP protein expression showed no difference between SB203580-treated cells and SB203580-TMPyP4 co-treated groups (*P* > 0.05). These results indicated that p38 MAPK activation was involved in TMPyP4-induced apoptosis in human cervical cancer cells.

## Discussion

TMPyP4, serving as a photosensitizer in PDT, has proved to play an essential role in controlling osteosarcoma [11]. Besides, TMPyP4 has been recently developed as a chemotherapeutics drug to inhibit telomerase activity through binding to and stabilizing telomeric G-quadruplex DNA in cancer cells [7–9]. Through directly interaction with telomeres, TMPyP4 can rapidly evoke antiproliferative effects [12]. In vivo, after intratumor injection of 10 mg/kg TMPyP4 for 10 days, cervical tumor growth in nude mice was significantly inhibited without any injury to the skin and internal organs [13]. Therefore, in this study, we used this agent for investigation in cultured human cervical cancer cells to further evaluate its antitumor effects and underlying mechanism.

In the present study, treatment with various concentration of TMPyP4 significantly induced inhibition of proliferation in human cervical cancer cells. Previous report suggested that TMPyP4 could lead to progressive telomere shortening that eventually resulted in cancer cell apoptosis [6]. Mikami-Terao et al. ever demonstrated that treatment with 100 μM TMPyP4 significantly inhibited the growth of K562 leukemic cells, with decreases of cells in the G(1) phase and increases of those in the S and G(2)/M phases after 48 h [14]. In the following study, Mikami-Terao and his team found that TMPyP4 at doses of 10, 20, 50 or 100 μM significantly inhibited the growth of retinoblastoma cell lines, Y79 and WERI-Rb1 cells. In contrast, cell apoptosis was induced by TMPyP4 in a dose-dependent manner in both cell lines, which was associated with the increased expression of phosphorylated p53 (Ser46) protein and activation of MAPK [15]. TMPyP4 at concentrations of 3, 6, 15, 30 or 60 μM significantly inhibited the proliferation and motility of human ovarian carcinoma A2780 cells but suppressed the expression levels of minichromosome maintenance protein-2 (MCM2) and carbonic anhydrase IX (CA-IX) [16]. Additionally, TMPyP4 inhibited proliferation while induced apoptosis in colon cancer cells SW480 by the suppression of Wnt/β-catenin signaling pathway [17]. More recently, a study demonstrated that, TMPyP4 at low doses less than 0.5 μM could promote the migration of human lung cancer A549 cells, HeLa, osteosarcoma U2OS and SAOS2. In contrast, TMPyP4 at high-dose larger than 2 μM inhibited cell proliferation and induced cell apoptosis in these cells [18]. These findings above provided new insights into TMPyP4 which could be developed as a possible anticancer drug.

Numerous studies have indicated that the mitogen-activated protein kinase (MAPK) signaling pathway plays an important role in regulating cell proliferation, promoting cell cycle progression [19–21], and inducing resistance to radiotherapy and chemotherapy in tumor cells [22, 23]. In addition, p38 MAPK signaling cascade is a major pathway participating in the apoptotic pathway to suppress apoptosis of tumor cells [24, 25]. For instance, K562 leukemic cells treated with 100 μM TMPyP4 showed a decrease in c-Myc protein expression, and an obvious increase in the expression of p21 (CIP1), p57 (KIP2), p38 MAPK, c-Jun N-terminal kinase, and extracellular signal-regulated kinase [14]. Most interestingly, our data showed that TMPyP4 could promote the expression level of p-p38 MAPK in human cervical cancer cells, implicating p38 MAPK activation as a potential target for TMPyP4. Also, we provided the evidence that the p38 MAPK signaling was involved in TMPyP4-indcued changes in proliferation and apoptosis of human cervical cancer cells.

## Conclusions

In conclusion, we not only revealed a critical role for TMPyP4 in human cervical cancer cell proliferation and apoptosis, but also explored the molecular mechanisms by which TMPyP4 contributed to its antitumor effects. Up-regulation of the p38 MAPK signaling pathway by TMPyP4 could inhibit cell proliferation while promoted cell apoptosis in human cervical cancer cells. This study provided insight into the molecular mechanism of the antitumor effects of TMPyP4, indicating the p38 MAPK signaling might be a potential therapeutic target for TMPyP4 in human cervical cancer.
